# Online interventions to prevent mental health problems implemented in school settings: the perspectives from key stakeholders in Austria and Spain

**DOI:** 10.1093/eurpub/ckab039

**Published:** 2021-07-07

**Authors:** Michael Zeiler, Stefanie Kuso, Martina Nitsch, Monika Simek, Tanja Adamcik, Rocio Herrero, Ernestina Etchemendy, Adriana Mira, Elia Oliver, Megan Jones Bell, Andreas Karwautz, Gudrun Wagner, Rosa Maria Baños Rivera, Cristina Botella, Karin Waldherr

**Affiliations:** 1 Department for Child and Adolescent Psychiatry, Medical University of Vienna, Vienna, Austria; 2 Ferdinand Porsche FernFH-Distance Learning University of Applied Sciences, Wiener Neustadt, Austria; 3 CIBER Pathophysiology of Obesity and Nutrition (CB06/03), Carlos III, Institute of Health, Av. Monforte de Lemos, Madrid, Spain; 4 Department of Personality, Evaluation and Psychological Treatments, Facultad de Psicología, Universidad de Valencia, València, Spain; 5 Department of Basic and Clinical Psychology and Psychobiology, Universitat Jaume I, Castelló de la Plana, Spain; 6 Department of Pychology and Sociology, Universidad de Zaragoza. Facultad de Ciencias Sociales y Humanas., Calle Cdad. Escolar, Teruel, Spain

## Abstract

**Background:**

Schools are key settings for delivering mental illness prevention in adolescents. Data on stakeholders’ attitudes and factors relevant for the implementation of Internet-based prevention programmes are scarce.

**Methods:**

Stakeholders in the school setting from Austria and Spain were consulted. Potential facilitators (e.g. teachers and school psychologists) completed an online questionnaire (*N*=50), policy makers (e.g. representatives of the ministry of education and health professional associations) participated in semi-structured interviews (*N*=9) and pupils (*N*=29, 14–19 years) participated in focus groups. Thematic analysis was used to identify experiences with, attitudes and needs towards Internet-based prevention programmes, underserved groups, as well as barriers and facilitators for reach, adoption, implementation and maintenance.

**Results:**

Experiences with Internet-based prevention programmes were low across all stakeholder groups. Better reach of the target groups was seen as main advantage whereas lack of personal contact, privacy concerns, risk for misuse and potential stigmatization when implemented during school hours were regarded as disadvantages. Relevant needs towards Internet-based programmes involved attributes of the development process, general requirements for safety and performance, presentation of content, media/tools and contact options of online programmes. Positive attitudes of school staff, low effort for schools and compatibility to schools’ curriculum were seen as key factors for successful adoption and implementation. A sound implementation of the programme in the school routine and continued improvement could facilitate maintenance of online prevention initiatives in schools.

**Conclusions:**

Attitudes towards Internet-based mental illness prevention programmes in school settings are positive across all stakeholder groups. However, especially safety concerns have to be considered.

## Introduction

Effective and sustainable programmes for the prevention of mental health problems in adolescents are urgently needed as several European epidemiological studies revealed a high prevalence of mental health disorders and at-risk behaviours in this age group.^1–3^ Schools are regarded as key settings for the implementation of prevention programmes as they offer unique access to a diverse population of adolescents in their familiar learning environment.^4^ The efficacy of school-based mental illness prevention programmes has been shown by numerous systematic reviews and meta-analyses including programmes targeted at the prevention of depression and anxiety,^5^^,^^6^ substance abuse,^7^ suicidality^8^ and eating disorders.^9^ Internet-based approaches like computer-based prevention programmes and smartphone applications (apps) are especially suitable for delivering mental health interventions to adolescents since young people are digital natives.^10^ Two recently published meta-reviews^11^^,^^12^ provide support for the effectiveness of Internet-based approaches for the prevention and early intervention of mental health problems across different mental health dimensions (attention-deficit hyperactivity disorder, autism, anxiety, depression, post-traumatic stress disorder, psychosis and eating disorders) for adolescents. Despite these promising findings regarding their efficacy, most existing initiatives lack maintenance and large-scale dissemination after the initial funding period has ended.^13^^,^^14^ A recent review highlights the need for systematic implementation research and inclusion of stakeholders when implementing adolescent mental health interventions in a new setting or cultural context to close the science-to-practice gap.^15^

Internet-based prevention programmes might have benefits when implemented in school settings as they generally require less staff support, can be initially administered by teachers, can—although delivered in group settings—be tailored to individual needs and mental health risks and are more cost-effective than face-to-face interventions.^10^^,^^16^^,^^17^ On the other hand, potential barriers might include time constraints due to the respective school curriculum, missing guidelines focussing on mental illness prevention in schools, missing or low financial support from policy makers (PM) and unclear responsibilities,^18^ which are likely to affect Internet-based interventions in a similar way than face-to-face interventions. Although the WHO’s health promoting schools approach provides a useful and established framework for including health promoting interventions into school settings, this framework does not focus on mental health interventions.^19^ Other potential barriers specific to Internet-based interventions include low adherence, concerns about safety, greater risk for misinterpretations, feelings of impersonality and technical difficulties.^10^

Furthermore, schools provide a useful setting given many different stakeholders (e.g. teachers, principals, pupils, parents, school psychologists, school physicians, ministry of education and other PM) must collaborate when initiating preventive interventions. Involving stakeholders in the early phase of programme development and adoption appear crucial for its success.^14^^,^^20^ To date, few studies have addressed different stakeholders’ perspectives in the development and implementation of mental health promotion and mental illness prevention programmes in school settings and none are found that explicitly focussed on Internet-based approaches. The perceived and actual needs of teachers, pupils and other school staff as well as the expected benefits of mental health services and prevention are regarded as relevant factors when adopting prevention programmes in schools.^4^^,^^21^^,^^22^ In addition, the attitudes towards mental illness prevention programmes, acceptability and experiences with these programmes as well as support from school principals, school staff and pupils are reported as highly relevant.^4^^,^^14^^,^^23^^,^^24^ Several programme characteristics of Internet delivery including high usability, the possibility to tailor the programme content to individual needs of pupils, flexible use and adaptability as well as proved effectiveness and perceived user benefit might foster adoption and implementation.^4^^,^^14^^,^^21^^,^^23^^,^^24^ As for maintenance, implementation with minimal resources of costs and staff, integration of the programme into the schools’ curriculum, close stakeholder collaborations including governmental stakeholders and continuing feedback and adaptations appear to be key factors to success.^4^^,^^14^^,^^21^^,^^24^^,^^25^ Kuosmanen et al.^26^ conducted a stakeholder survey exploring the needs of students and staff towards online mental health programmes in an alternative education setting for early school leavers. From the students’ point of view, activity-based programmes, focus on practical skills, positive and encouraging content that is tailored to individuals’ needs as well as attractive layout are fostering factors to increase user engagement. From the staff’s perspective, careful planning and timetabling, flexibility in delivery and additional face-to-face support were found as the most relevant factors for successful implementation.^26^

From a public health perspective, the reach of a large number of adolescents who are willing to participate in a prevention programme, a high willingness of schools to offer interventions to their pupils as well as high feasibility of implementation and maintenance of preventive interventions in school settings are—apart from effectiveness—crucial for intervention success and to gain high population impact.^27^ As found in a recent systematic review, these factors are understudied and under-reported in the current literature.^28^ We also have reports that Internet-based preventive interventions for adolescents were associated with low participation rates and high dropout,^29^ low adoption by schools^30^ and missing sustainability.^31^ Thus, more research is needed on how to improve reach, adoption, implementation and maintenance of such interventions in the school setting.

To our knowledge, this is the first study which addressed stakeholders’ experiences with, attitudes and needs regarding Internet-based mental illness prevention programmes as well as factors relevant for reach, adoption, implementation and maintenance in the school setting. The study is part of the European project ‘ICare’, which aimed at implementing several Internet-based interventions targeting common mental health problems for various target groups (TGs) in different settings (healthcare system, school and university) across six European countries.^32^ In the first phase of ICare, a stakeholder survey was conducted which investigated factors relevant for implementation, dissemination and exploitation of ICare interventions in healthcare systems, schools and universities in Europe.

## Methods

### Design and instruments

According to the design of the ICare stakeholder survey described in Nitsch et al.^33^ in this supplement, we consulted three different stakeholder groups in the school setting: (i) potential facilitators (teachers, school psychologists, school physicians, school social workers and pupils elected to represent other pupils at their school boards), (ii) PM (e.g. ministry of education, official national representatives of school psychologists and school physicians and official national representatives of pupils in political bodies) and (iii) the TG of ICare interventions implemented in the school setting (pupils aged 14–19 years at Austrian and Spanish schools irrespective of their mental health status). Due to the specific characteristics of each stakeholder group, we applied different survey methods. We used an online questionnaire for potential facilitators because we wanted to reach a large number of participants, semi-structured in-person or telephone interviews with PM as individuals in high positions of school authorities are more likely to participate in interviews than in questionnaires, and focus groups with the TG as the interaction in a group is considered as a crucial factor to generate deeper insights into attitudes relevant for the intervention success.^33^ The results of this concurrent mixed-methods design were synthesized during analysis.^33^ Potential facilitators and representatives of the TG were recruited from different schools in Austria and Spain. The items of the online questionnaire as well as the topic guides for the focus groups and semi-structured interviews (see electronic Supplementary material) were developed by the ICare consortium,^33^ and reflect the dimensions Reach, Adoption, Implementation and Maintenance of the RE-AIM framework.^27^ The online questionnaire had 27 overall questions and obtained both quantitative information (e.g. ratings on a 10-point scale regarding the relevance of different prevention topics and characteristics of Internet-based programmes) and qualitative information [e.g. open-ended questions regarding (dis)advantages of online programmes and fostering/hindering factors for the RE-AIM dimensions].

Main themes that were addressed included (i) experiences with Internet-based interventions to prevent mental health disorders implemented in the school setting, (ii) attitudes towards online mental illness prevention including presumed advantages and disadvantages, (iii) groups that are considered underserved and (iv) needs (covering overarching aims, topics and characteristics) as well as (v) hindering and fostering factors and context parameters for reach, adoption, implementation and maintenance in connection with online programmes to prevent mental health problems in the school setting. Fostering and hindering factors for the effectiveness dimension of the RE-AIM model were not targeted in this study as this is part of an upcoming RCT.^34^ In the online questionnaire as well as in the interviews and focus groups, all participants were provided with definitions of ‘prevention’, ‘Internet-based interventions’ as well as a definition of each RE-AIM dimension to ensure a common understanding of these terms across all participants (see electronic Supplementary material for details).

### Recruitment and procedure

Different stakeholders in the school setting in Austria and Spain were approached following a criterion based sampling strategy described in Nitsch et al.^33^ In Austria, we informed potential facilitators about the project via e-mail and asked them to complete the online questionnaire. Furthermore, they were asked to distribute the online questionnaire in their school community. Contact information (e-mail addresses) was extracted from a random sample of websites of different types of secondary schools in all regions of Austria. In Spain, stakeholders were contacted via e-mail, informed about the project and asked to complete the online questionnaire. Contact information was obtained through the local government in Valencia with its previous consent. The recruitment strategies in Austria and Spain differed because in Spain the stakeholders’ contact details were not publicly available.

PM were recruited via e-mail or telephone requests and invited to take part in a semi-structured interview. Depending on their availability and time, the interviews were conducted in-person or on the phone. Interview duration ranged from 32 to 61 min (mean: 43.3, SD: 9.7). Informed consent for audiotaping the interview was provided by the participants.

We approached pupils aged 14–19 years from different schools for the focus groups. This age range was chosen because one of the ICare interventions, ‘Healthy Teens @ School’,^34^ is targeted to pupils of this age group. Pupils were recruited from different schools and youth organizations. In Austria, focus groups were held in the rooms of the schools or the youth organization, while in Spain the focus groups were held at university premises. Informed consents were obtained from all focus group participants including consent for audio recording. Additionally, for pupils younger than 18 years, consent from a legal representative was obtained. The duration of the focus groups ranged from 25 to 75 min (mean: 55.2, SD: 26.2). In Austria, focus group participants received a €10-gift card for their participation. Spanish participants received no compensation. Ethical approval for this study was obtained from the Ethics Committee of the Medical University of Vienna (EK Nr. 2209/2015), the General Ethics Committee of Vienna (EK Nr. 16-006-VK), the Ethics Committee of the University of Valencia (H1453976699999) and the University Jaume I (5/2017).

### Data analyses

Focus groups and interviews were transcribed verbatim in German and Spanish language. Spanish transcripts and answers to open-ended questions from the Spanish online questionnaire were translated into German or English by researchers. Both the transcripts and the answers to the open-ended questions of the online questionnaire were coded and organized in NVivo 11 Pro software.^35^ Thematic analysis was used to organize and analyze the identified themes related to the research questions.^36^ In detail, the coding and analyses process was performed as follows: two researchers (S.K. and M.S.) coded the qualitative data from the focus groups and interviews. To ensure common understanding about the coding process the researchers coded three interviews/focus groups together, rather than developing a code book. The remaining interviews/focus groups were coded independently by the two researchers and the codes were cross-checked afterwards. Subsequently, the two researchers jointly interpreted the data and identified themes as well as relevant subthemes. A combination of deductive and inductive approach was chosen: the main categories (e.g. reach, adoption, implementation and maintenance) were deduced from the research questions, subthemes were identified from the data. The themes and subthemes were further discussed within the research team (S.K., M.S., M.N., M.Z. and K.W.) and iteratively adapted until consensus was reached. Initially, this process was done separately for the focus groups and interviews. The open-ended questions of the online questionnaire mostly consisted of catchwords or short phrases. They were coded subsequently, and the resulting categories matched the themes/subthemes emerged from the analysis of the interview and focus group transcripts. Finally, the results were merged by exploring similarities and differences between stakeholder groups. Generally, the emerging themes did not differ or contradict between countries and stakeholder groups, thus, the results are presented across countries and stakeholder groups. Only if a theme was discussed in just one stakeholder group or differences were apparent, it is highlighted in the text.

Quantitative items of the online questionnaire included ratings to assess the extent of experiences with Internet-based prevention programmes in addition to the degree of relevance regarding different needs and characteristics of these programmes. These were analyzed at the item level by using descriptive statistics (percentages, mean, SD and median).

## Results

In total, we obtained 50 online questionnaires from potential facilitators, and conducted nine semi-structured interviews with PM and five focus groups involving a total of 29 pupils (3–8 per group) in both countries. The main characteristics of the sample are presented in table 1.

**Table 1:** Sample characteristics of included stakeholders of the school setting

Online questionnaire (total *N* =50)	
Number of individuals per country	*N* (%)
Austria Spain	41 (82.0) 9 (18.0)
Function	*N* (%)
Pupils’ representative	4 (8.0)
Teacher	29 (58.0)
School physician	7 (14.0)
School psychologist	3 (6.0)
School social worker	4 (8.0%)
Other	3 (6.0)
Years of experiences in their function	Mean (SD)/median (range)
Years	14.89 (10.41)/13 (1–33)
Semi-structured interviews (total *N* =9)	
Number of interviews per country	*N* (%)
Austria	5 (55.6)
Spain	4 (44.4)
Type of interview	*N* (%)
In-person	7 (77.8)
Telephone	2 (22.2)
Participants per gender	*N* (%)
Females	5 (55.6)
Males	4 (44.4)
Sector^a^	*N* (%)
Governing sector	6 (66.7)
Insurance (school service point)	1 (11.1)
School health care provider	2 (22.2)
Official representation of target group	1 (11.1)
Years of experience in their function	*N* (%)
0–5 years	4 (44.4)
6–10 years	0 (0.0)
11–20 years	2 (22.2)
>20 years	2 (22.2)
Unknown	1 (11.1)
Focus groups (total *N* =5)	
Number of focus groups per country	*N* (%)
Austria	3 (60.0)
Spain	2 (40.0)
Participants per sex	*N* (%)
Total	29 (100)
Females	18 (62.1)
Males	11 (37.9)

a As one individual has overlapping functions, the numbers and percentages do not sum up to N = 9 and 100%.

### Experiences

PM stated to have no or little experiences with online prevention programmes in mental health. Only one participant reported experiences with a pilot project regarding a blended intervention on self-esteem, which was not institutionalized in their setting. Focus group participants from the TG mentioned experiences with prevention in general. However, they also considered support from friends, family and from persons within the school system as mental illness prevention. They did not have any experiences with online prevention programmes specifically but talked about health-related apps, social media or psychological self-tests in this context. About 48% of potential facilitators (F) who participated in the online survey, affirmed the statement ‘I have read or heard about Internet-based prevention programmes in the field of mental health’, only 4.3% has already looked into and 2.2% has implemented such programmes in the school setting. Nobody had used online prevention programmes or guided their implementation. On a 10-point scale (0=no experiences at all, 10=a lot of experience), the estimated level of experience was rated as extremely low (mean: 0.52, SD=1.43, median =0).

### Attitudes

Participants were asked what they consider as the most important advantages and disadvantages of online prevention programmes in the field of mental health. Potential facilitators had the possibility to indicate three advantages and disadvantages in an open-ended question of the online questionnaire. Overall, five major advantages and three disadvantages were identified.

Advantage 1: low structural barriers. The use of online prevention programmes can help to overcome physical or practical barriers to mental health services with regard to time, location and costs. They can be delivered at low or no costs (‘cheaper for the healthcare system’, F) and ‘anytime and anywhere’ (PM).

Advantage 2: low inhibition threshold/psychological barriers. The anonymity related to online programmes is perceived as an asset, when it comes to facilitate factors of the utilization of mental health services for teenagers, ‘especially for those who would not turn to a psychologist or psychiatrist’ (F).

I can imagine that this is for all people with difficulties talking to people when they look at them. I know a few people who prefer talking in the dark, because then they don’t have to look at someone, and then you don’t see, for example, when people cry. This is easier to handle over the Internet. (TG)

Advantage 3: information/promotion of mental health literacy. The dissemination of objective information on mental health to the youth population is seen as contribution to destigmatize mental illness in society because information on this topic can help ‘to normalise and reduce shame’ (TG) and a visible high number of programme participants ‘shows that you are not alone with a problem’ (TG). Furthermore, it could help teenagers to realize when they are at risk for developing mental health problems and encourage them to reach out for help. Online programmes in this regard are seen as ‘a way to start, so that the children see that there are possibilities to get help’ (PM).

Advantage 4: familiarity of young people with technology. Since young people have little reservations towards using new technologies in their everyday life, it is seen as a good channel to reach them. This theme was only brought up by the facilitators (e.g. teachers and school psychologists). ‘Using a medium that is part of everyday life for young people’ (F).

Advantage 5: increased reach. The reduction of practical and psychological barriers facilitates the access to Internet-based prevention programmes for a larger number of pupils and the reachability of former underserved subgroups. ‘I think with Internet-based programmes you can simply reach much, much more children and teenagers than with face-to-face contacts’ (PM).

Disadvantage 1: lack of personal contact. The absence of personal contact and face-to-face interaction was not only seen as advantage. Participants also mentioned some possible pitfalls. Personal face-to-face contact is considered crucial to build trust and relationships, especially in the field of mental health. For example, if someone ‘[…]never sees the person face-to-face, the body language is missing, all of that gets lost over the Internet’ (PM) and online programmes ‘fail to convey empathy’ (F). Moreover, it is assumed that pupils are more likely to drop out or not stick to a programme, compared to regular face-to-face meetings, because they feel less obliged towards an online programme than towards a person since ‘you don’t have the same obligation as when you talk to someone in person about a problem’(PM). Also, one interviewee mentioned that participants would ‘take it more seriously if it were not online’ (TG). Finally, without personal contact and real-time communication, misunderstandings could occur more easily, which could lead to wrong diagnoses and hinder ‘adequate provision of help in a crisis situation’ (F). When pupils already have mental health problems, or such problems become obvious while using an online programme, health professionals have less possibility to offer help or track the progression of their problems. Thus, some participants argued that online programmes might not be sufficient for more severe mental health problems: ‘It might have its pitfalls, when problems pop up, how does the teenager deal with that? When a problem comes up during this programme, how can we react to it?’ (PM)

Disadvantage 2: digital technology and Internet. Five subthemes were identified that refer to the use of digital technologies. Some stakeholders raised doubts regarding their (i) privacy because ‘it is always claimed, that in the Internet everything gets saved and maybe some people find it discomforting, when they can be traced forever’ (TG). Furthermore, (ii) online programmes are only available for teenagers with access to the Internet. The pupils raised concerns that (iii) if a forum was part of the programme, others might misuse it by posting unserious comments or making fun of it. However, they also mentioned that access via login and a moderator could counteract that problem. (iv) The effectiveness of prevention via digital technologies is doubted, especially as they are associated with higher dropout. Finally, (v) overuse of digital technologies among teenagers was discussed. Some potential facilitators stated that the younger generations already spend too much time online and this should not be further encouraged.

Disadvantage 3: implementation during school time. Concerning implementation and integration in the school context, stigmatization and group dynamics came up as key issues in the focus groups with pupils. The fact that other pupils could observe their peers while using the programme, could lead to bias or social desirability because ‘at home you would be more honest than at school, where you know someone could read along’ (TG).

Some participants raised doubts that especially among teenagers, not all pupils in a class will take the programme seriously and unfavourable group dynamics might occur. Therefore, it might be better to promote and introduce the programme in schools and give the pupils the opportunity to use it at home.

I would give everyone in class a code at the beginning of the school year and give a short introduction, but not push it. Because the people who need it will come forward anyway, and otherwise it will rather backfire, half of the class will make fun of it and be like, ‘funny, we don’t need that, we are way to cool for that’. (TG)

In the online survey, the facilitators were asked to weigh advantages and disadvantages of Internet-based prevention programmes compared to face-to-face contacts on a 10-point scale (−5=much more disadvantages, +5=much more advantages, 0=neutral). According to this stakeholder group, advantages slightly outweighed disadvantages (mean=1.08, SD=2.58, median=1). Furthermore, the extent to which they would be in ‘favour of integrating online mental health preventive interventions’ in the school setting (mean=6.62, SD=2.90, median=7) and to which they would actively support the integration of online interventions (mean=6.52, SD=2.99, median=7) were rated as medium to high (rated on a 10-point scale: 0=not at all, 10=absolutely).

### Underserved groups

With respect to underserved groups regarding mental illness prevention, all stakeholder groups discussed that pupils at risk for mental health problems and with subclinical symptoms would benefit most from Internet-based prevention programmes. They referred to pupils with specific sociodemographic risk factors including gender and age, family background (single parent families, low parental educational background and migration background) and specific psychological problems (see also needs). While focus group participants believed that pupils who are highly motivated to improve their health would benefit most, PM discussed that pupils who are performing badly in school would profit most. Furthermore, introverted and shy pupils who would not participate in face-to-face interventions were regarded as underserved and pupils who know someone suffering from mental health problems would also benefit from psychoeducational programmes. However, several stakeholders brought up that all adolescents should be targeted as this age group is generally at risk for developing mental health problems.

### Needs

Regarding overarching aims that online prevention programmes should achieve, three main topics were identified: (i) promotion of mental health, (ii) providing information about mental health issues to increase awareness and sensitize for this topic, and thereby contribute to destigmatization of mental health problems and (iii) referring to professionals if needed. In the online questionnaire, facilitators were asked to rate the importance of different aims on a 10-point scale (0=not at all important, 10=very important) including the prevention of disease onset, reduction of risk factors, increase of protective factors, prevention of progression of first symptoms and the reduction of the severity of first symptoms. All aims were regarded as almost equally important (mean ratings between 6.1 and 6.7).

**Figure 1:**
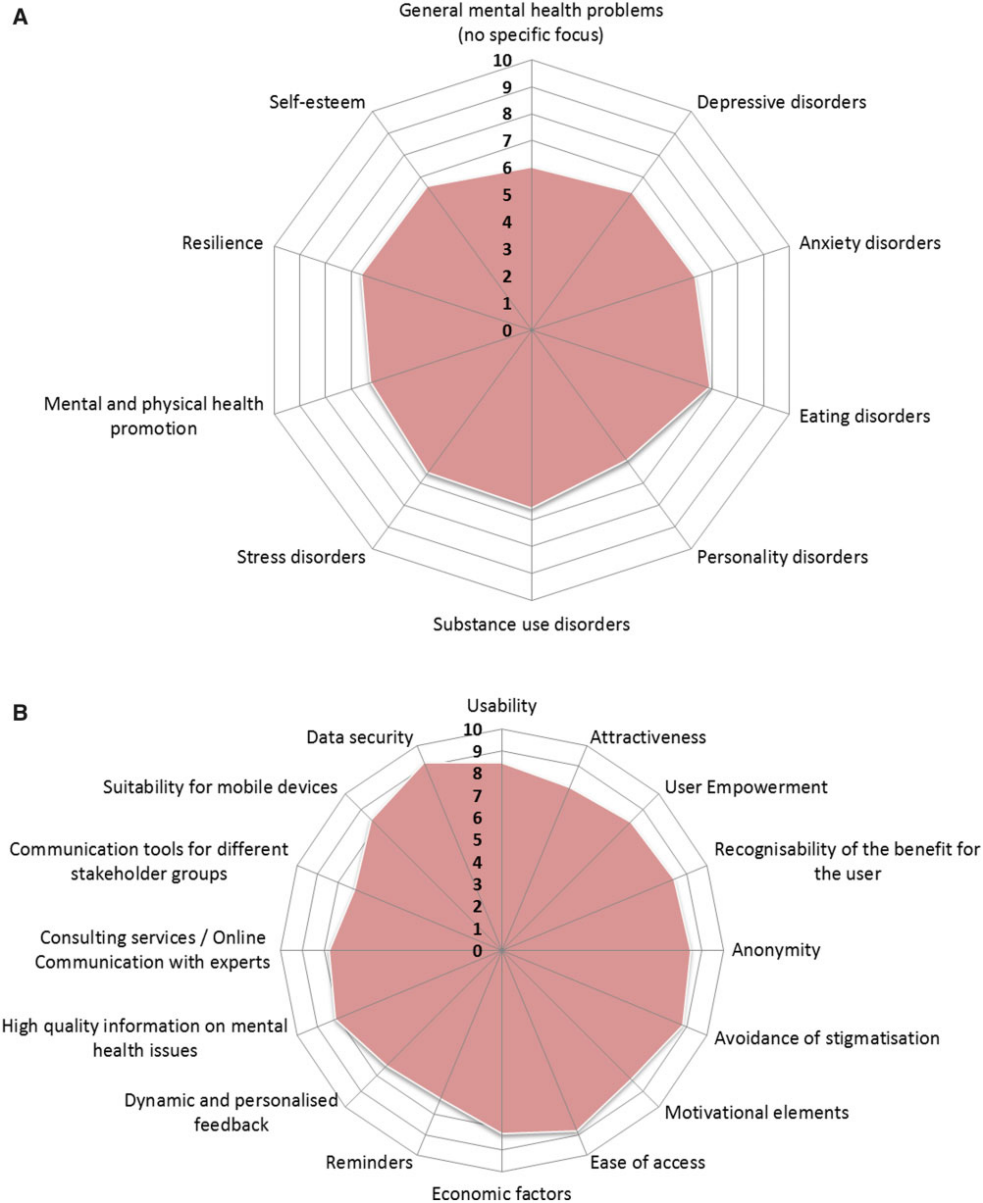
Mean ratings of facilitators regarding the relevance of (a) topics and (b) characteristics of Internet-based prevention programmes (0=not at all relevant, 10=very relevant)

According to pupils and PM, online prevention programmes should focus on (i) mental disorders including anxiety disorders, depression, drug abuse and addiction, eating disorders and mental disorders in general; (ii) acute or chronic conditions associated with mental health problems including stress, family problems, bullying and sexual abuse and (iii) skills and healthy lifestyle including general health, learning and motivation, resilience, love and sexuality, nutrition, exercise and social skills. In the online questionnaire, potential facilitators rated the relevance for suggested topics on a 10-point scale (0=not at all relevant, 10=very relevant). The mean ratings are depicted in figure 1a.

Stakeholders discussed how an online prevention programme in mental health should look like and which ‘characteristics’ it should have. Five overarching themes were identified.

1. Attributes of development process: this theme includes elements that should be considered while developing the programme. (i) Participation of pupils and teachers’ in the development process was seen as imperative to assure that the programme is appropriate for the TG. (ii) A certification guaranteeing the credibility and quality of a programme as last step in the development process prior to dissemination was mentioned as crucial, since there are a lot of dubious offers on the Internet, which are hard to evaluate.

2. General requirements: besides layout and content issues, four major characteristics were reported. (i) Anonymity and data protection are crucial especially in the field of mental health.

On the Internet there has to be some kind of warranty that it is anonymous and data are not forwarded to third parties. Because this is difficult, when you make your problems public. (TG)

When writing about a problem, I would like to know: Will this be recorded? Will it be deleted? Or will it be kept and used for some purpose. […] Because once this gets hacked, it is not anonymous anymore. (TG)

Other important basic requirements are (ii) usability, which means that the programme must be easy to access and to use, (iii) responsive web-design (particularly suitable for mobile phones) and (iv) provision of the programme in different languages.

3. Presentation of content: This theme describes how the content is communicated to the users. Participants pointed out, that (i) a programme should be TG-appropriate. An online programme for teenagers should be rather concise and not contain too much text. It should be gamified and interactive and give users the opportunity to choose for themselves if they want to read more about special topics and to keep their attention. (ii) A positive framing or wording and the avoidance of pathologizing language were mentioned as important issues. Since a programme about mental health needs to convey a sense of trust, it should also give the impression of (iii) reliability and confidentiality. Furthermore, (iv) an attractive layout and design is important for the users.

4. Media and tools: media used, to provide programme content has to be diverse to increase motivation and keep users interested. This includes media like pictures and videos as well as tools like embedded exercises, case examples for identification, search bars, self-assessments and follow-ups. Advertising should be avoided.

5. Contact options: Another important characteristic that was discussed were contact options, (i) with other users or peers via chats, forums or social networks in order to talk to other people with similar problems; (ii) with professionals within the programme, so that users who have problems or questions besides the programme content can contact professionals directly via e-mail, chat or skype; (iii) blended approaches, the combination of face-to-face and online sessions, were also mentioned as possible options.

Additionally, stakeholders’ needs are described in the context of the RE-AIM results below when they were mentioned in the context of reach, adoption, implementation or maintenance of Internet-based prevention programmes.

In the online questionnaire, the facilitators were asked to rate the relevance of suggested characteristics for online mental illness prevention programmes on a 10-point scale (0=not at all relevant, 10=very relevant). The mean ratings per characteristic are depicted in figure 1b. All characteristics were regarded as moderate to very relevant whereby data security was rated most relevant (mean>9).

## Influencing factors following the RE-AIM dimensions

Stakeholders were also asked about presumed fostering and hindering factors for reach, adoption, implementation and maintenance of online mental health prevention programmes in schools.

### Reach

The mentioned themes concerning factors relevant to reach a high number of adolescents can be broken down into three subsequent steps: (1.) inform potential users about the programme, (2.) get them to log in and (3.) underlying usage conditions.

1. Information about the programme. Relevant factors are (i) support by multiplicators like teachers, parents, older pupils and peers and (ii) different modes of promotion in schools such as posters, a link on the school website or possibilities to get an introduction during class or test the programme at school.

2. Getting users to log in. Participants’ views were mixed concerning the registration process: while access via registration and login is perceived as psychological barrier (‘I don’t think it’s good if I have to enter my e-mail address’, TG), it might increase quality and prevent misuse of the programme (‘Without registration, every other person will misuse it’, TG).

3. Underlying usage conditions. Concerning underlying usage conditions of the programme, the central theme was to make it TG-appropriate. This refers to its characteristics, which are described above, e.g. the use of different media like videos and pictures. While using the programme during school hours would increase reach, this was also seen sceptical. First, this would make participation virtually obligate. Second, pupils might not feel comfortable working on a mental health programme in a classroom because their privacy is restricted, and therefore, they might fear stigmatization. Third, group dynamics in schools could lead to negative effects, such as not taking the programme seriously and making fun of it.

### Adoption

Adoption refers to the number of schools and school staff who are willing to initiate a prevention programme. The topics were sorted into three categories, depending on whether or how much they can be influenced by providers.

1. Modifiable factors*.* These include providing the schools and teachers with (i) information about the programme, the significance of prevention and data protection; (ii) keeping the costs for the schools low and (iii) keeping the efforts for the schools and teachers low by providing support, deciding on responsible persons and keeping the additional workload low (‘It is always about the organisational, timely resources, financial resources.’, PM).

2. Attitudes. These are only partly influenceable and include (i) support of the principal and the teachers, (ii) openness for innovation and (iii) the perceived need for mental health prevention.

If the school […] sees a demand, that such a project could be useful for the pupils; so, if the school feels that pupils need it. In principle, if the school is open – and I mean, there are different school cultures, of course – but if the school is open for innovations or projects, because there are schools which already do or did a lot and don’t want to get another thing imposed. (PM)

3. Given circumstances. This refers to (i) the technical infrastructure of schools, (ii) the temporal limitations due to the school’s schedule, (iii) existing (competing) programmes that may hinder the adoption of new programmes and (iv) whether the school is participating in the WHO health promoting school framework,^32^ which is a network of schools who agreed to strengthen their capacity as a healthy setting for living, learning and working and may facilitate the adoption of new initiatives related to mental health.

### Implementation

Implementation means that the intervention can be delivered in the school setting as intended. Important fostering factors for implementation are according to stakeholders (i) personal support by the provider, (ii) simplicity of the programme and (iii) the implementation by specialized staff like school physicians or school psychologists instead of teachers.

It is important to show teachers that it needs a specific procedure, because I assume that it is somehow evidence-based. Teachers tend to modify things according to their experience and pick some pieces of the puzzle […] I think it is easier with school physicians or school psychologists. They have more background knowledge of what evidence-based means in that context. To be honest, we never managed to accomplish something as intended. They leave or add parts (laughs). (PM)

Furthermore, the programme has to (iv) ‘fit into the annual schedule and curriculum of the school’ (‘There is no extra lesson provided for this.’, F).

### Maintenance

Concerning the extent to which a programme becomes part of the routine school practises and policies, the identified topics can be differentiated into supportive structures and quality of the programme.

1. Supportive structures. This includes (i) ongoing efforts to ensure reach, (ii) secured financing and (iii) a sound implementation in schools, which includes a responsible person in school, making the programme part of the school routine and an agenda in faculty meetings and/or part of a mental health focus (‘[…] for example, all pupils of the first and fifth class should automatically receive the programme’, F; ‘[…] it is regularly discussed in class’, PM).

2. Quality of the programme. Ideally, the pupils (i) are satisfied with the programme and have a ‘positive attitude’ towards it (‘The most important factor is that everyone who is part of the school community accepts it and commits to it., PM), and (ii) recognize the benefits of using the programme (‘It is a barrier […] if it is just associated with additional workload and pupils don’t see any changes’, F). Furthermore, (iii) ‘continuous improvement and evaluation’ of the programme based on user participation and feedback are seen as crucial factors for maintenance.

## Discussion

This study aimed to assess the experiences, attitudes and needs of different stakeholder groups of the Austrian and Spanish school setting towards Internet-based prevention programmes in the field of mental health and to identify factors relevant for reach, adoption, implementation and maintenance.

Experiences regarding the use and implementation of online prevention in mental health in school settings were very low across all stakeholder groups. This indicates that such programmes are not yet widespread in the school setting and that both potential implementers and users might not have concrete ideas how Internet-based prevention in mental health may look like and may work in practice. Given that current literature emphasizes that the implementers’ practical experiences with the use of online prevention programmes and (prior) experiences of success are important for gaining positive attitudes towards the programme as well as for adoption and implementation,^14^^,^^18^ this has important practical implications: Prior to offering the programme to the TGs, implementers in schools should be provided with in depth-theoretical explanations and should be given the possibility to gain practical experience with the programme content and how the programme is delivered. Indeed, expanding the stakeholders' knowledge about a prevention programme has been found to increase implementation fidelity and programme sustainability even in stakeholder groups with initially limited expertise in the field,^37^ which indicates that this phase of programme implementation should not be neglected.

Although experiences were low, stakeholder groups expressed quite positive attitudes towards Internet-based programmes and their implementation in the school setting and mentioned various potential advantages including lower practical barriers, such as service times, regional availability of services and low costs, and lower psychological barriers through anonymity and the absence of face-to-face contact. These advantages of Internet-based prevention were also discussed in previous studies.^10^^,^^26^ However, considering the advantages of anonymity on the one hand (which was emphasized by the TG) and disadvantages of the lack of personal contact on the other hand it seems to be difficult to find the right balance in programme development. While a maximum of anonymity might increase initial programme participation, lack of personal contact is known to decrease individual adherence and increase the risk of dropout,^12^ which may be especially problematic for adolescents with more pronounced symptoms of mental health problems. The use of blended interventions combing face-to-face sessions and supporting online tools,^38^ implementing personal guidance and support^29^ as well as the use of stepped-care approaches^39^ (e.g. online programmes for low risk students or students with mild mental health problems with optional personal contact to mental health professionals; face-to-face sessions for students with more severe symptoms) may mitigate these concerns. Furthermore, when approaching schools for Internet-based preventive initiatives, the potential advantages of these types of programmes but also their limitations should be emphasized and openly discussed.

Several underserved groups that should be approached to participate in online prevention programmes were mentioned by the stakeholders reflecting different prevention steps from universal prevention (all adolescents), to selected prevention (adolescents with elevated risk for mental health problems due to specific sociodemographic characteristics) and indicated prevention (adolescents with subclinical mental health disorders). Whereas most previous preventive initiatives (both, face-to-face and online interventions) did not differentiate risk levels of adolescents, but used uniform approaches only (either one universal programme for all students or one programme for specific risk groups only),^6^^,^^12^^,^^28^ this points to tailored prevention approaches as the future of prevention allowing different programme versions based on individual risks.^40^ This would ease the implementation of Internet-based interventions, particularly in a school setting, and would be an advantage compared to face-to-face interventions.

Stakeholders mentioned numerous topics and characteristics of online prevention programmes, which they regarded as relevant and which have direct impact on the development, design and implementation of online mental illness prevention programmes in school settings. Relevant topics were not restricted to common mental health issues like anxiety and depression but also included healthy lifestyle issues like learning skills, social skills and nutrition. This finding indicates that broader prevention approaches (e.g. including content to promote life skills) may be beneficial in terms of engagement than approaches targeting the prevention of specific symptoms of psychiatric disorders. The majority of characteristics identified in this study overlap with characteristics found in another stakeholder survey^26^ and are closely connected to the identified advantages and disadvantages. For example, privacy concerns can be tackled via information on how data are protected and the lack of personal contact could be addressed by including online communication tools. An overarching topic that was discussed from various angles was stigmatization and destigmatization, a topic that has not gained much attention so far. The use of online programmes is seen as an advantage in providing psychoeducation to large groups and in destigmatization of mental health problems but they might also be stigmatizing for pupils when implemented during school hours (e.g. fear of being observed while working on the programme, negative group dynamics). In a recently published systematic review on Internet-based prevention for eating disorders,^28^ all researchers have used the school setting to reach adolescents and include them into their online programme, but in half of the studies the programme was not implemented during school hours. We lack available research on the practical implications of programme outcomes as identified by stakeholders. For example, although the impact on adherence has not been evaluated so far, using the school setting for recruitment but not for implementation might reduce stigmatization. Moreover, providing a tailored programme to all pupils in a school class instead of approaching pupils at risk only may counteract negative group dynamics and feelings of stigmatization, and might in this respect be superior to face-to-face programmes. These aspects should be addressed in future research.

Close and on-going collaborations between programme developers, mental health professionals (e.g. school psychologists) and school staff were discussed as highly relevant throughout all programme phases, including programme development; for instance, by providing accurate information about the programme and how to use it (adoption), by supporting teachers with expert knowledge (implementation) and by evaluating and adapting the programme continuously (maintenance). Whereas the training of school staff was seen as an important prerequisite of programme implementation in prior studies,^23^^,^^25^ the results of this study indicate that (mental) health professionals should be strongly involved in programme delivery. This finding corresponds to the results of a meta-analysis showing that intervention effects were slightly larger when external staff (such as health professionals) delivered the programme compared to school staff.^6^

Regarding the adoption of online prevention programmes, tight schedules, limited technical infrastructure and competing projects have to be taken into account as potential structural barriers, while positive attitudes of school staff towards mental illness prevention and the perceived necessity of these programmes can facilitate adoption. These results confirm the finding of previous studies highlighting the relevance of these factors for adoption.^4^^,^^24^ In practice, this means that investing a reasonable amount of time for providing information about the efficacy of online programmes to prevent mental problems and describing the benefits for schools is of utmost importance for their successful dissemination.

Regarding maintenance, different studies emphasize the importance of embedding online prevention programmes into the school routine and curriculum.^4^^,^^21^^,^^25^ This was also confirmed in this study where some participants discussed that the programme should be part of an overarching mental health focus in schools, which can be further developed and evaluated on a regular basis. Along with other mentioned requirements for sustainable programmes, such as secured financing, stable positive attitudes towards the programme and recognition of user benefit, the long-term implementation of Internet-based prevention programmes in mental health in the school setting still remains the main challenge.^13^^,^^14^

### Limitations

Stakeholders’ prior experiences with Internet-based prevention programmes in the field of mental health were very low. Thus, the results of this study cannot be generalized to more experienced stakeholders. Although all stakeholder groups received a definition of prevention programmes (programmes to prevent onset of mental health problems, to increase protective factors, to reduce risk factors or to reduce symptoms of mental health problems), most participants discussed programmes targeted at adolescents with advanced symptoms of mental health problems. This indicates that the results might not be fully generalizable to universal prevention purposes. Although we provided corresponding definitions, some stakeholders found it difficult to differentiate between the RE-AIM dimensions adoption, implementation and maintenance when discussing fostering and hindering factors. This might be due to the fact that the RE-AIM phases build on each other and the same context factors may be relevant for different phases. In Spain, only nine potential facilitators could be recruited to participate in the online questionnaire, which was less than expected. Thus, we were not able to analyze country differences.

## Conclusion

This study provides useful insights into factors relevant for the design and sustainable implementation of Internet-based programmes for the prevention of mental health problems in school settings in Europe. Although the experiences with online mental illness prevention programmes were very low across all stakeholder groups, attitudes towards Internet-based prevention were quite positive, which is encouraging and an important prerequisite for a successful adoption and implementation. However, a couple of doubts and potential barriers raised by the stakeholders have to be considered. Although PM support the implementation of Internet-based prevention programmes, guidelines and policies on how mental illness prevention initiatives can be embedded in school settings are scarce. Accordingly, future research should focus on the translation of research findings into policy making and practice, which is an essential requirement for the maintenance of online prevention programmes.

## Supplementary data

Supplementary data are available at *EURPUB* online.

## Supplementary Material

ckab039_Supplementary_Material
